# Effects of ergothioneine supplementation on meiotic competence and porcine oocyte development

**DOI:** 10.14202/vetworld.2024.1748-1752

**Published:** 2024-08-13

**Authors:** Megumi Nagahara, Zhao Namula, Qingyi Lin, Koki Takebayashi, Nanaka Torigoe, Bin Liu, Fuminori Tanihara, Takeshige Otoi, Maki Hirata

**Affiliations:** 1 Bio-Innovation Research Center, Tokushima University, Tokushima, Japan; 2 Department of Animal Reproduction, Faculty of Bioscience and Bioindustry, Tokushima University, Tokushima, Japan; 3 Department of Animal Reproduction, College of Coastal Agricultural Sciences, Guangdong Ocean University, Zhanjiang, China

**Keywords:** antioxidant, DNA fragmentation, ergothioneine, maturation, porcine oocytes

## Abstract

**Background and Aim:**

The antioxidant effects of ergothioneine (EGT) on *in vitro* culture of porcine zygote are not well established. The study investigated the impact of EGT supplementation on meiotic competence and development of porcine oocytes.

**Materials and Methods:**

The impact of EGT concentrations (0, 5, 10, 25, 50, and 100 μM) during *in vitro* maturation (IVM) on the progression of meiotic maturation, fertilization, and blastocyst formation in porcine oocytes was assessed. The DNA fragmentation level was evaluated to determine oocyte and blastocyst quality.

**Results:**

The proportion of metaphase II oocytes was significantly greater (p < 0.05) in EGT-cultured oocytes than in control oocytes, regardless of the EGT concentration, and those oocytes with 10 μM or more EGT had fewer DNA-fragmented nuclei (p < 0.05). Blastocysts derived from oocytes cultured with 10 μM EGT had the highest proportion (p < 0.05), while those from control oocytes or oocytes cultured with 50 μM or less EGT had significantly higher proportions. Despite EGT supplementation, there were no noticeable differences in total cell numbers and DNA fragmentation indices in the derived blastocysts.

**Conclusion:**

Supplementing with EGT during IVM leads to better oocyte maturation, quality, and embryonic development due to decreased DNA fragmentation. The present study failed to elucidate the mechanism of DNA fragmentation reduction by EGT. More research needs to be conducted to explore the antioxidant mechanism of EGT.

## Introduction

*In vitro* culture exposes oocytes and embryos to oxidative stress caused by reactive oxygen species (ROS) due to the lack of inherent protective antioxidant mechanisms *in vivo*, leading to both structural and functional impairments [1]. Damage to DNA, RNA, and proteins by ROS-induced inhibition potentially compromises oocyte maturation, fertilization, and embryonic development through processes such as apoptosis and necrosis [2, 3]. Moreover, the exposure of oocytes to ROS results in meiotic arrest and increased apoptosis of oocytes [4]. It has been reported that supplementation with antioxidants, such as β-mercaptoethanol [5], chlorogenic acid [6], and curcumin [7], reduces ROS-induced oxidative stress and improves oocyte meiosis and embryonic development.

A dietary compound called Ergothioneine (EGT), sourced exclusively from specialty mushrooms, is gaining recognition for its antioxidant properties [8, 9]. EGT protects against the events observed during atherogenesis by preventing inflammation-induced expression of adhesion molecules [10]. The transporter of EGT, which is highly concentrated in mitochondria, specifically protects DNA and other cells from oxidative damage due to mitochondrial production of superoxide radicals [11]. Despite extensive research, the antioxidant effects of EGT on porcine oocyte development and apoptosis remain unclear.

This study examined the impact of EGT supplementation on oocyte maturation, embryo development, and the quality of both oocytes and embryos during *in vitro* maturation (IVM).

## Materials and Methods

### Ethical approval

No live animals were used in this study, so no ethical approval was required. Ovaries of prepubertal crossbred gilts were collected from a local slaughterhouse (Nippon Food Packer, Japan) after slaughtering of the gilts.

### Study period and location

The data collection for this study was conducted from January to February 2024 at Bio-Innovation Center, Tokushima University, Japan.

### IVM

IVM of oocytes was performed according to the methods described by Namula *et al*. [7] with minor modification by adding different antioxidants of EGT. Briefly, ovaries of prepubertal crossbred gilts (Landrace × Large White × Duroc) were collected from a local slaughterhouse. Cumulus-oocyte complexes (COCs) were collected from follicles (3–6 mm in diameter) using a surgical blade. Only COCs with uniform dark-pigmented ooplasm and intact cumulus cell mass were selected for this experiment. Approximately 50 COCs were cultured in maturation medium consisting of tissue culture medium 199 with Earle’s salts (TCM 199; Thermo Fisher Scientific, Waltham, MA, USA) supplemented with 10% (v/v) porcine follicular fluid, 50 μM sodium pyruvate (Sigma-Aldrich, St. Louis, MO, USA), 2 mg/mL D-sorbitol (Fujifilm Wako Pure Chemical Corporation, Osaka, Japan), 10 IU/mL equine chorionic gonadotropin (Kyoritsu Seiyaku, Tokyo, Japan), 10 IU/mL human chorionic gonadotropin (Kyoritsu Seiyaku), and 50 μg/mL gentamicin (Sigma-Aldrich) for 22 h in four-well dishes (Nunc A/S, Roskilde, Denmark). The COCs were subsequently transferred to maturation medium without hormone supplementation and cultured for an additional 22 h. The COCs were incubated at 39°C in a humidified incubator containing 5% CO_2_. To assess the effect of EGT supplementation on the meiotic competence and DNA integrity of oocytes, as well as on fertilization and blastocyst formation, COCs were cultured in IVM medium supplemented with 0 (control), 5, 10, 25, 50, and 100 μM of EGT for a total of 44 h.

### Analysis of nuclear oocyte maturation and DNA fragmentation

To determine nuclear maturation and DNA fragmentation, the oocytes were fixed after IVM and analyzed using a combined technique of simultaneous nuclear staining and terminal deoxynucleotidyl transferase nick-end labeling (TUNEL), according to procedures previously described by Thongkittidilok *et al*. [12]. Briefly, oocytes were denuded from cumulus cells using 150 IU of hyaluronidase and mechanical pipetting. Denuded oocytes were fixed in 4% paraformaldehyde at 4°C overnight and subsequently permeabilized with 0.1% Triton-X100 for 1 h at room temperature (25°C). Next, they were incubated at 4°C overnight in phosphate-buffered saline containing 10 mg/mL bovine serum albumin. The oocytes were then incubated in fluorescein-conjugated 2-deoxyuridine-5-triphosphate and TUNEL reagent (Roche Diagnostics Corp., Tokyo, Japan) for 1 h at 38 C. After TUNEL staining, oocytes were counterstained with 1 μg/mL 4′, 6-diamidino-2-phenylindole (DAPI; Invitrogen Co., Carlsbad, CA, USA) for 10 min. The sections were then treated with an anti-bleaching solution (Slow-Fade; Molecular Probes Inc., Eugene, OR, USA), mounted on a glass slide, and sealed with clear nail polish. The labeled oocytes were examined using an epifluorescence microscope (Eclipse 80i; Nikon, Tokyo, Japan) equipped with epifluorescence illumination. The oocytes were determined to be either in the germinal vesicle, condensed chromatin, metaphase I, anaphase I–telophase I, or metaphase II (MII) stage according to the chromatin configuration based on DAPI staining. To assess DNA damage in all oocytes after IVM, the nuclei labeled with TUNEL were counted.

### *In vitro* fertilization (IVF) and assessment

After IVM, oocytes were washed with porcine fertilization medium (PFM; Research Institute for the Functional Peptides Co., Yamagata, Japan), and approximately 50 oocytes were transferred to a four-well dish containing 500 μL of PFM containing frozen-thawed spermatozoa at a final concentration of 1 × 10^6^ sperm/mL and co-cultured for 5 h at 39°C in a humidified incubator containing 5% CO_2_, 5% O_2_, and 90% N_2_ in the air. To evaluate fertilization, some oocytes were fixed for 10 h after insemination with acetic acid: Ethanol (1:3 v/v) for 48–72 h. Fixed oocytes were stained with acetic orcein (1% orcein in 45% acetic acid) and examined using phase-contrast microscopy. Oocytes containing both female and male pronuclei were considered fertilized and categorized as normal or polyspermic based on the number of swollen sperm heads and/or pronuclei in the cytoplasm [7]. The proportion of monospermic fertilization was calculated by dividing the number of monospermic fertilized oocytes by the number of fertilized oocytes.

### *In vitro* culture and assessment

After IVF, the COCs were washed with porcine zygote medium (PZM-5; Research Institute for the Functional Peptides Co.), transferred to four-well dishes containing 500 μL of PZM-5, and subsequently incubated for 72 h at 39°C in a humidified incubator with an atmosphere of 5% CO_2_, 5% O_2_, and 90% N_2_. Only cleaved embryos were selected 72 h after IVF and transferred to a porcine blastocyst medium (Research Institute for the Functional Peptides Co.). The embryos were then cultured for an additional 4 days to assess their development into blastocysts. DNA fragmentation was evaluated after blastocyst formation using the TUNEL assay, as described above. DNA fragmentation indices were calculated by dividing the number of cells containing DNA-fragmented nuclei (labeled with TUNEL) by the total number of cells.

### Statistical analysis

The experiments were repeated 8 times for EGT-treated oocytes. Statistical significance was inferred by analysis of variance, followed by Fisher’s protected least significant difference (PLSD) test using STATVIEW (Abacus Concepts, Inc., Berkeley, CA, USA). The percentage data were subjected to arcsine transformation before statistical analysis. Data are expressed as the mean ± standard error of the mean. Differences with a value of p < 0.05 were considered significant.

## Results

As shown in Table-1, when the oocytes were cultured with EGT, the proportion of oocytes that reached MII was significantly increased compared with the control oocytes cultured without EGT, irrespective of the EGT concentration (p < 0.05). The proportion of oocytes with DNA-fragmented nuclei was significantly lower (p < 0.05) in oocytes cultured with 10 μM or more EGT than in control oocytes. Furthermore, the proportion of total fertilization was significantly higher in oocytes cultured with 25 μM EGT than in control oocytes (p < 0.05). There were no significant differences in the proportion of monospermic fertilization among the groups.

**Table-1:** Effect of EGT supplementation on the maturation and fertilization of porcine oocytes *in vitro*.

Concentration of EGT (µM)	No. of examined oocytes	No. (%) of oocytes with*		No. (%) of oocytes with DNA-fragmented nucleus	No. of examined oocytes	No. (%) of oocytes	
	
		GVBD	MII			Fertilized	Monospermy
Control	114	105 (92.3 ± 2.3)^c^	68 (58.4 ± 3.3)^c^	4 (3.8 ± 1.5)^a^	105	63 (60.6 ± 3.8)^b^	43 (68.5 ± 4.3)
5	106	100 (94.8 ± 2.0)^a,b,c^	74 (70.3 ± 3.2)^b^	2 (2.0 ± 1.3)^a,b^	102	67 (66.5 ± 3.9)^a,b^	49 (73.4 ± 2.1)
10	112	111 (99.2 ± 0.8)^a^	89 (79.1 ± 3.8)^a^	0 (0)^b^	105	71 (68.2 ± 2.9)^a,b^	49 (70.1 ± 2.5)
25	127	124 (97.6 ± 1.6)^a,b^	91 (71.9 ± 2.3)^a,b^	0 (0)^b^	102	71 (71.1 ± 4.5)^a^	49 (71.1 ± 3.6)
50	110	105 (95.2 ± 2.1)^a,b,c^	80 (73.4 ± 3.3)^a,b^	1 (0.7 ± 0.7)^b^	106	67 (63.7 ± 2.9)^a,b^	49 (72.2 ± 6.6)
100	104	98 (94.3 ± 1.8)^b,c^	72 (68.4 ± 3.6)^b^	1 (0.7 ± 0.7)^b^	104	68 (66.3 ± 3.8)^a,b^	47 (70.4 ± 4.3)

* GVBD=Germinal vesicle breakdown, MII=Metaphase II, EGT=Ergothioneine. ^a-c^Values with different superscript letters in the same column are significantly different (p < 0.05)

As shown in Table-2, the proportions of blastocysts derived from oocytes cultured with 50 μM or less EGT were significantly higher (p < 0.05) than those from control oocytes, with the highest proportion at 10 μM EGT. However, no significant differences were observed in the proportions of cleavage, total cell numbers, and DNA fragmentation indices of the resulting blastocysts, irrespective of EGT supplementation.

**Table-2:** Effect of EGT supplementation during* in vitro* maturation on the development and DNA integrity of porcine embryos.

Concentration of EGT (µM)	No. of examined oocytes	No. (%) of embryos		Total cell numbers of blastocyst	DNA fragmentation indices (%)

		Cleaved	Developed for blastocysts		
Control	273	224 (82.1 ± 1.9)	20 (7.4 ± 0.8)^d^	34.2 ± 1.0	3.1 ± 0.8
5	262	216 (82.3 ± 2.9)	35 (13.5 ± 2.4)^b,c^	38.5 ± 2.5	1.6 ± 0.4
10	249	210 (84.2 ± 1.5)	47 (18.9 ± 1.7)^a^	37.9 ± 1.9	2.0 ± 0.4
25	248	202 (82.2 ± 2.1)	31 (13.1 ± 1.9)^b,c^	39.5 ± 3.3	2.2 ± 0.5
50	266	222 (83.5 ± 1.4)	43 (16.1 ± 2.2)^a,b^	34.7 ± 1.4	2.8 ± 0.8
100	268	216 (80.9 ± 2.2)	26 (9.7 ± 1.0)^c,d^	34.0 ± 2.1	2.4 ± 0.6

^a-d^Values with different superscript letters in the same column are significantly different (p < 0.05). EGT=Ergothioneine

## Discussion

Supplementing the *in vitro* culture medium with antioxidants enhances meiotic maturation of oocytes and blastocyst formation in mammals due to its distinctiveness from the *in vivo* environment [13]. 10 μM EGT addition to IVM medium significantly increased oocyte maturation and blastocyst formation rates. 10 μM EGT or more led to decreased DNA fragmentation in mature oocytes. These observations indicate that EGT supplementation improves the meiotic competence of oocytes and embryonic development potential after IVF by reducing DNA damage during IVM culture. It has been reported that EGT possesses potent endogenous anti-hydroxyl (OH), anti-peroxyl, and anti-peroxynitrite radical antioxidant activities compared with classical antioxidant molecules such as glutathione, Trolox, and uric acid [14]. In addition, EGT plays a significant role in providing robust protection against metal-induced oxidative damage *in vivo* by forming complexes with copper ions [15, 16]. The robust antioxidative efficacy of EGT is derived from its accelerated redox reaction rate compared with other thiols [15]. *In vivo*, EGT promptly and directly mitigates OH radicals, which are pivotal initiators of chain reactions, thereby shielding lipids from peroxidation. This reactivity toward OH radicals surpasses that of amino acids and DNA [17, 18], thereby endowing EGT with the capability to safeguard proteins and DNA. Our previous study also demonstrated that supplementation with 50 μM chlorogenic acid improved maturation, fertilization, and blastocyst formation rates and reduced DNA fragmentation rates in porcine oocytes exposed to oxidative stress induced by H_2_O_2_ during maturation culture [6]. Similarly, in the present study, we observed that the antioxidant activity of EGT enhanced oocyte development after IVF. This is consistent with previous reports demonstrating that antioxidants protect porcine oocytes against oxidative stress and play an important role in the acquisition of post-fertilization development [6, 7, 19].

## Conclusion

10 μM EGT supplementation during IVM improves meiotic maturation and blastocyst formation of porcine oocytes by decreasing DNA damage. The present study failed to explain how EGT reduces DNA fragmentation. More research is needed to understand the antioxidant properties of EGT fully.

## Data Availability

The supplementary data can be available from the corresponding author on a reasonable request.

## Authors’ Contributions

MN and MH: Conceptualization and writing – original draft and – review and editing. MN and ZN: Investigation and methodology. QL, KT, NT, and BL: Investigation and formal analysis. TO: Project administration, supervision, and writing – review and editing. FT: Supervision and writing – review and editing. All the authors have read, reviewed, and approved the final manuscript.
